# Genetically Encoded Green Fluorescent Ca^2+^ Indicators with Improved Detectability for Neuronal Ca^2+^ Signals

**DOI:** 10.1371/journal.pone.0051286

**Published:** 2012-12-11

**Authors:** Masamichi Ohkura, Takuya Sasaki, Junko Sadakari, Keiko Gengyo-Ando, Yuko Kagawa-Nagamura, Chiaki Kobayashi, Yuji Ikegaya, Junichi Nakai

**Affiliations:** 1 Brain Science Institute, Saitama University, Saitama, Japan; 2 Laboratory of Chemical Pharmacology, Graduate School of Pharmaceutical Sciences, University of Tokyo, Tokyo, Japan; Western University of Health Sciences, United States of America

## Abstract

Imaging the activities of individual neurons with genetically encoded Ca^2+^ indicators (GECIs) is a promising method for understanding neuronal network functions. Here, we report GECIs with improved neuronal Ca^2+^ signal detectability, termed G-CaMP6 and G-CaMP8. Compared to a series of existing G-CaMPs, G-CaMP6 showed fairly high sensitivity and rapid kinetics, both of which are suitable properties for detecting subtle and fast neuronal activities. G-CaMP8 showed a greater signal (*F*_max_/*F*_min_ = 38) than G-CaMP6 and demonstrated kinetics similar to those of G-CaMP6. Both GECIs could detect individual spikes from pyramidal neurons of cultured hippocampal slices or acute cortical slices with 100% detection rates, demonstrating their superior performance to existing GECIs. Because G-CaMP6 showed a higher sensitivity and brighter baseline fluorescence than G-CaMP8 in a cellular environment, we applied G-CaMP6 for Ca^2+^ imaging of dendritic spines, the putative postsynaptic sites. By expressing a G-CaMP6-actin fusion protein for the spines in hippocampal CA3 pyramidal neurons and electrically stimulating the granule cells of the dentate gyrus, which innervate CA3 pyramidal neurons, we found that sub-threshold stimulation triggered small Ca^2+^ responses in a limited number of spines with a low response rate in active spines, whereas supra-threshold stimulation triggered large fluorescence responses in virtually all of the spines with a 100% activity rate.

## Introduction

Understanding brain function requires techniques for monitoring the spatio-temporal activity patterns of individual neurons and synapses. A promising approach for this purpose is Ca^2+^ imaging that can detect neuronal events as a change in Ca^2+^ fluorescence intensity. Recently, Ca^2+^ imaging using green fluorescent protein (GFP)-based genetically encoded Ca^2+^ indicators (GECIs) has been introduced as an alternative to using chemically synthesized fluorescent Ca^2+^ indicators [1]–[6]. GECIs offer two remarkable advantages over synthesized indicators: (i) GECIs can be targeted to specific cell types and specific subcellular compartments [7]–[10], and (ii) GECIs are applicable to long-term expression (over months) [4], [11]–[13]. Although GECIs have improved, there remains a need for GECIs with greater signals and more rapid kinetics to allow the reliable detection of individual neuronal spikes.

In this study, we developed high-sensitivity and fast-responsivity GECIs, termed G-CaMP6 and G-CaMP8, by mutagenizing existing G-CaMPs. These novel indicators allow us to reliably monitor neural spikes with larger fluorescence signals and higher temporal resolution than G-CaMP3, a recently reported variant of G-CaMP2 [4]. We also demonstrate that G-CaMP6-actin, a fusion protein of G-CaMP6 and actin, can be used to image spine-specific Ca^2+^ signals in response to presynaptic single spikes at the single-synapse level.

## Results

### Development of Improved G-CaMPs by Site-directed and Random Mutagenesis

**Figure 1:**
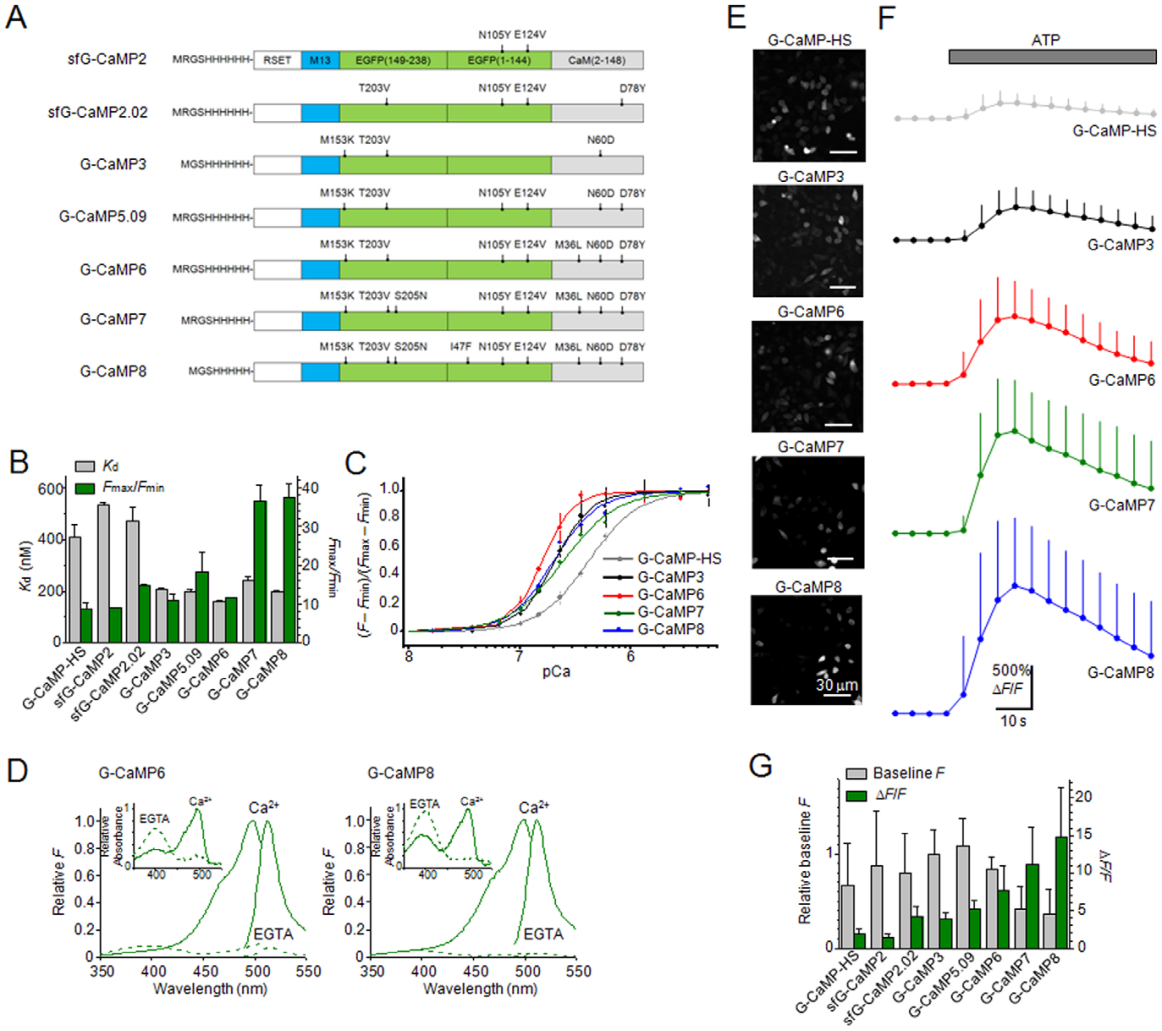
Characterization of G-CaMPs *in vitro* and in HeLa cells. **A**, Schematic representation. Mutations are indicated with respect to G-CaMP2. RSET and M13 are tags that encode hexahistidine and a target peptide for Ca^2+^-bound CaM derived from MLCK, respectively. The amino-acid numbers of EGFP and CaM are indicated in parentheses. **B**, Dynamic range (*F*_max_/*F*_min_) and Ca^2+^ affinity (*K*_d_). Error bars, s.d. (*n* = 3 each). **C**, Ca^2+^ titration curve. Curves were fit according to the Hill equation. *K*_d_ is shown in **B**. **D**, Normalized fluorescence and absorbance (inset) spectra of G-CaMP6 and G-CaMP8 in 1 µM Ca^2+^ or 1 mM EGTA. **E**, Fluorescence images of HeLa cells expressing G-CaMPs. Bars, 30 µm. **F**, Time course of the changes (Δ*F*/*F*) in G-CaMP fluorescence in response to 100 µM ATP. Error bars, s.d. **G**, Baseline fluorescence and peak responses (Δ*F*/*F*) to ATP application in HeLa cells.

In an effort to create a superior GECI, we first introduced mutations from “superfast GFP” [14], which was recently reported to enhance the folding activity of GFP, into a prototype GECI, G-CaMP2 [15], because some known folding mutations improve the functionality of GECIs [16], [17]. Through screening, we found that a G-CaMP2 variant with two mutations (N105Y and E124V) introduced into the circularly permutated enhanced GFP (EGFP) domain, termed sfG-CaMP2 (Fig. 1A), showed a greater dynamic range (*F*_max_/*F*_min_ = 9.03±0.06, *n* = 3) than G-CaMP2 [15] (*F*_max_/*F*_min_ = 4.8) (Fig. 1B). For further improvement, mutations known to stabilize the chromophore [i.e., T203V in the circularly permutated EGFP domain and D78Y in the calmodulin (CaM) domain] were introduced into sfG-CaMP2 [18], and this variant was termed sfG-CaMP2.02 (Fig. 1A). sfG-CaMP2.02 showed a greater signal increase (*F*_max_/*F*_min_ = 14.8±0.28, *n* = 3) than sfG-CaMP2 (Fig. 1B). Subsequently, mutations from G-CaMP3 [4] were introduced into sfG-CaMP2.02 to examine whether certain mutations could further improve the functionality of GECIs. Among the variants of sfG-CaMP2.02, we identified a superior variant termed G-CaMP5.09 (Fig. 1A), which showed not only a high signal (*F*_max_/*F*_min_ = 18.4±4.94, *n* = 3) but also a high affinity for Ca^2+^ (*K*_d_ = 200±7.0 nM, *n* = 3) (Fig. 1B and C). G-CaMP5.09 differs from G-CaMP2.02 by M153K in the circularly permutated EGFP domain and N60D in the CaM domain (Fig. 1A). Next, we attempted to enhance the Ca^2+^ sensitivity of G-CaMP5.09 to improve the detection of weak Ca^2+^ signals. For this purpose, we introduced mutations known to modify the affinity of CaM for myosin light chain kinase (MLCK) [19] because G-CaMP Ca^2+^ sensitivity is based on the intramolecular interaction between the CaM domain and the M13 domain of MLCK. As expected, G-CaMP6, a variant of G-CaMP5.09 bearing an M36L substitution in the CaM domain (Fig. 1A), showed a higher Ca^2+^ affinity (*K*_d_ = 158±4.0 nM, *n* = 3) than G-CaMP5.09 or the previously reported G-CaMP2 variants G-CaMP-HS [17] and G-CaMP3 [4] (Fig. 1B and C). The dynamic range of G-CaMP6 (*F*_max_/*F*_min_ = 11.4±0.11, *n* = 3) was not significantly different from that of G-CaMP3 (*F*_max_/*F*_min_ = 11.1±1.59, *n* = 3) (Fig. 1B). The spectra of G-CaMP6 were similar to those of G-CaMP2, with the exception that the Ca^2+^ absorbance peak (498 nm) was red-shifted by ∼10 nm (Fig. 1D). Next we performed random mutagenesis on G-CaMP6 by using an error-prone PCR [15] and were able to screen a highly responsive variant termed G-CaMP7, which differs from G-CaMP6 by a deletion of histidine (ΔH) in the RSET domain and an S205N mutation in the circularly permutated EGFP domain (Fig. 1A). The dynamic range of G-CaMP7 (*F*_max_/*F*_min_ = 36.6±4.10, *n* = 3) was ∼3-fold greater than that of G-CaMP6, even though this variant showed a lower Ca^2+^ affinity (*K*_d_ = 243±14 nM, *n* = 3) than G-CaMP6 (Fig. 1B and C). By performing further random mutagenesis on G-CaMP7, we obtained a more sensitive variant of G-CaMP7 termed G-CaMP8, bearing a deletion of arginine (ΔR2) in the RSET domain and an I47F mutation in the circularly permutated EGFP domain (Fig. 1A). It was intriguing that the ΔR2 mutation reported in G-CaMP3 [4] was incidentally incorporated into G-CaMP8. The Ca^2+^ affinity of G-CaMP8 (*K*_d_ = 200±1.1 nM, *n* = 3) was between those of G-CaMP6 and G-CaMP7 and similar to that of G-CaMP3 (*K*_d_ = 205±5.0 nM, *n* = 3) (Fig. 1B and C). The spectra of G-CaMP8 were similar to those of G-CaMP6 (Fig. 1D). To assess the functionality of the GECIs in a cellular environment, we next expressed the G-CaMPs in HeLa cells. The baseline fluorescence (Fig. 1E) and the response to ATP stimulation (Δ*F*/*F*) (Fig. 1F) of each G-CaMP variant are summarized in Figure 1G. As expected from their dynamic ranges and Ca^2+^ affinities (Fig. 1B and C), the signal amplitude (Δ*F*/*F*) of G-CaMP6, G-CaMP7 and G-CaMP8 was greater than that of G-CaMP3. The baseline fluorescence of G-CaMP7 and G-CaMP8, unlike G-CaMP6 and other variants, was lower than that of G-CaMP3.

### Comparison of G-CaMPs in Pyramidal Neurons

**Figure 2:**
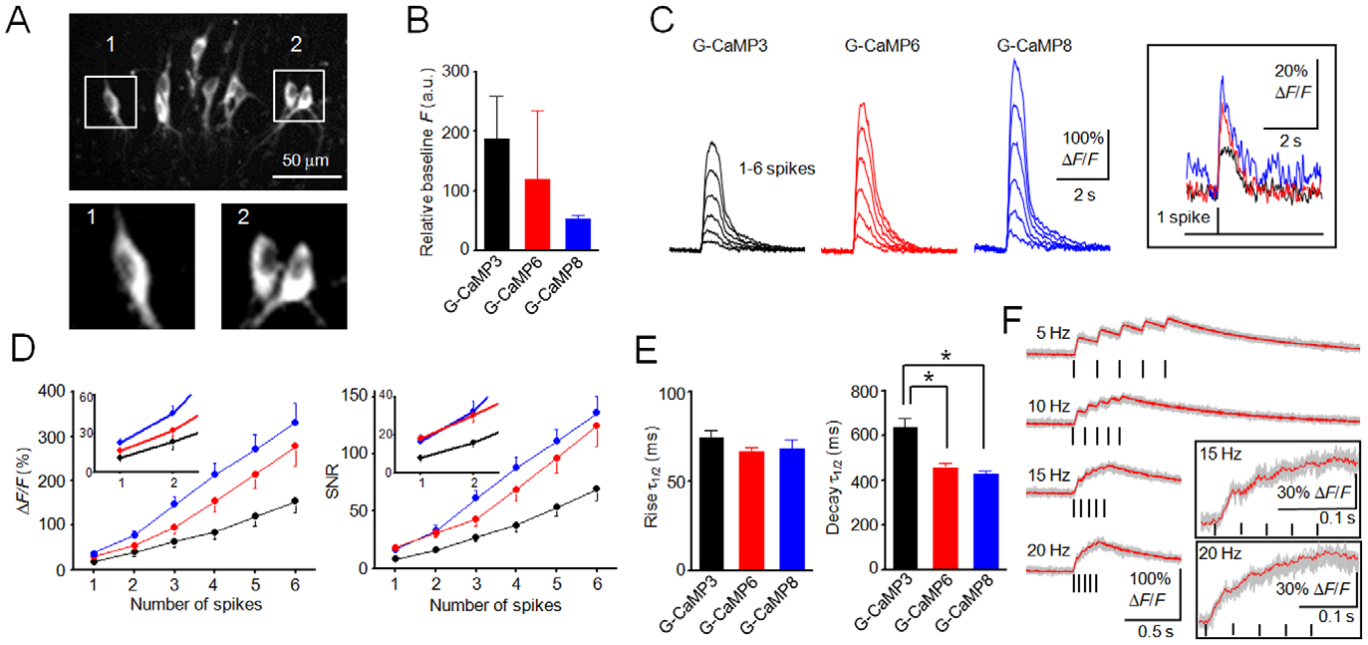
Characterization of G-CaMPs in cultured hippocampal slices. **A**, Expression of G-CaMP6 in hippocampal CA3 pyramidal neurons. Inset: Higher-magnification views are shown in the bottom panels. **B**, Baseline fluorescence of hippocampal neurons expressing G-CaMP3, G-CaMP6 and G-CaMP8. No significant differences in variance were detected among the three groups (*P*>0.05, χ^2^ = 2.90, Bartlett’s test). Error bars, s.d. (*n* = 7 each, *P*>0.05, Tukey’s test). **C**, Representative traces of the response (Δ*F*/*F*) to spike trains. The frequency of stimuli was 50 Hz. Right: Magnified views of single spikes. **D**, Mean responses (Δ*F*/*F*) and SNRs of G-CaMP3 (black), G-CaMP6 (red) and G-CaMP8 (blue). Inset: Magnified views of 1–2 spikes. Error bars, s.e.m. (*n* = 7 each). **E**, Rise and decay time constants for the responses to single spikes. Error bars, s.e.m. (*n* = 7 each; *P<0.05 in Tukey’s post-hoc test following one-way ANOVA). **F**, Trial-averaged responses of G-CaMP6 to spike trains. Gray, individual traces (*n* = 10 trials); red, averaged traces. Bars indicate stimulus timing. Inset: Magnified views.

We next evaluated the performance of G-CaMPs in pyramidal neurons in cultured rat hippocampal slices. In the cultured slices, the expression of G-CaMPs and mCherry was driven by the CMV promoter following transfection of the cells with the construct via targeted single-cell electroporation [20]. Simultaneous patch-clamp recording and confocal Ca^2+^ imaging were performed on G-CaMP-expressing neurons 24–48 h after electroporation. The baseline fluorescence of the neurons expressing G-CaMP6 (Fig. 2A) was similar to that of neurons expressing G-CaMP3, whereas G-CaMP8-expressing neurons exhibited lower fluorescence intensity than those expressing the other G-CaMPs (Fig. 2B). To monitor spike-induced Ca^2+^ responses, the neurons were current-injected to evoke 1–6 spikes at a frequency of 50 Hz. All experiments were carried out at room temperature (25–28°C), unless otherwise specified. G-CaMP6 and G-CaMP8 responded to single spikes with 100% probability. The Δ*F*/*F* amplitudes of Ca^2+^ transients evoked by single spikes were 17.4±3.5%, 27.9±4.5% and 37.8±5.2%, and the signal-to-noise ratios (SNRs) were 8.0±1.5, 18.3±1.5 and 16.4±3.5 for G-CaMP3, G-CaMP6 and G-CaMP8, respectively (Fig. 2C and D; *n* = 7 each). The signal amplitudes grew almost linearly as the spike number increased (Fig. 2C and D). Over the entire stimulus range, the amplitudes of the Ca^2+^ transients and the SNRs of G-CaMP6 and G-CaMP8 were consistently higher than those of G-CaMP3. The rise time of the spike-induced Ca^2+^ transients did not differ among G-CaMP3, G-CaMP6 and G-CaMP8 (*P*>0.05, Tukey’s test). On the other hand, the signal decay of G-CaMP6 and G-CaMP8 was significantly faster than that of G-CaMP3 (G-CaMP3, decay τ_1/2_ = 638±38 ms; G-CaMP6, decay τ_1/2_ = 457±20 ms; G-CaMP8, decay τ_1/2_ = 428±11 ms; Tukey’s test; *n* = 7 each) (Fig. 2E). The rapid kinetics and the fairly high Ca^2+^ sensitivity (Fig. 1B and C) of G-CaMP6 contributed to an increased temporal resolution of the signals within spike trains up to 15–20 Hz (Fig. 2F).

**Figure 3:**
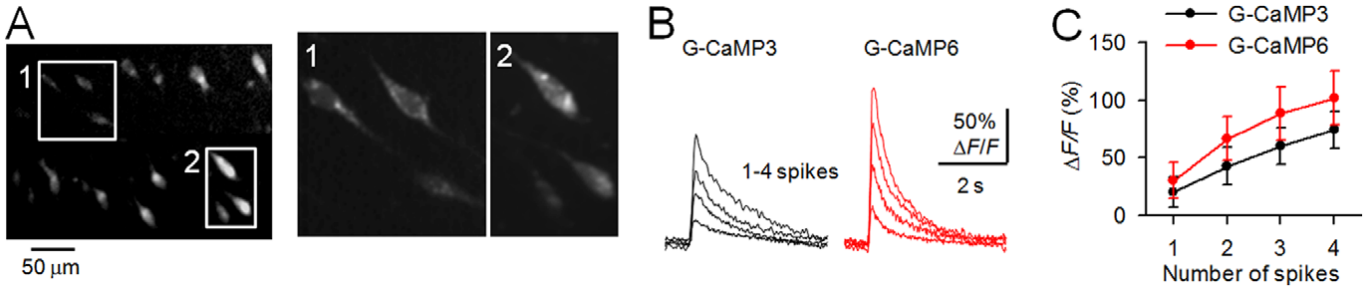
Comparison of G-CaMP responses in acute cortical slices. **A**, Confocal image of G-CaMP6-expressing cortical pyramidal cells. The expression of G-CaMP6 was driven by the CAG promoter via *in utero* plasmid electroporation. Inset: Higher-magnification views are shown in the right panels. **B**, Representative Δ*F*/*F* traces in response to 1–4 spikes evoked at 50 Hz. **C**, Mean responses (Δ*F*/*F*) of G-CaMP3 and G-CaMP6 to spike trains. Error bars, s.e.m. (G-CaMP3, *n* = 4 cells; G-CaMP6, *n* = 5 cells).

The detectability of G-CaMPs was also evaluated in pyramidal neurons in acute cortical slices. The expression of G-CaMPs in the mouse brain was driven by *in utero* electroporation, as previously described [21]. Consistent with the results presented in Figure 2C and D, G-CaMP6 performed better than G-CaMP3 in acute cortical slices prepared from mice at postnatal day 10–16 (Fig. 3). This result also implies that G-CaMP6 can be stably expressed in neurons for at least 4 weeks.

**Figure 4:**
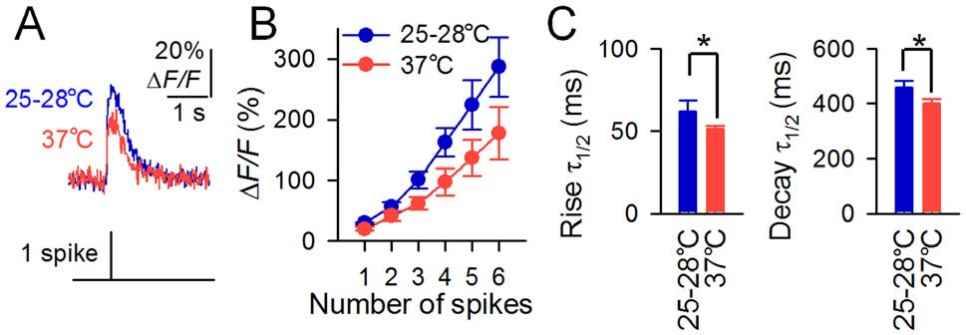
Temperature dependence of G-CaMP6 signals. **A**, Representative traces of the fluorescence response (Δ*F*/*F*) of G-CaMP6 to a single spike at 25–28°C and at 37°C. **B**, Mean responses (Δ*F*/*F*) of G-CaMP6 to spike trains. Error bars, s.e.m. (*n* = 6 each). **C**, Rise and decay time constants of the responses of G-CaMP6 to single spikes. (*P<0.05, paired *t*-test).

It is known that temperature significantly affects spike-dependent Ca^2+^ transients: they are small and short near physiological temperature because Ca^2+^ is removed quickly by strong Ca^2+^-pumping activity [22]. Here, the Δ*F*/*F* value of G-CaMP6 was ∼40% lower at 37°C than at 25–28°C (Fig. 4). The kinetics of G-CaMP6 were faster at 37°C (rise τ_1/2_ = 51.7±1.83 ms, decay τ_1/2_ = 402±15.1 ms, *n* = 6) than at 25–28°C (rise τ_1/2_ = 62.0±6.52 ms, decay τ_1/2_ = 458±23.7 ms, *n* = 6) (Fig. 2E).

**Figure 5:**
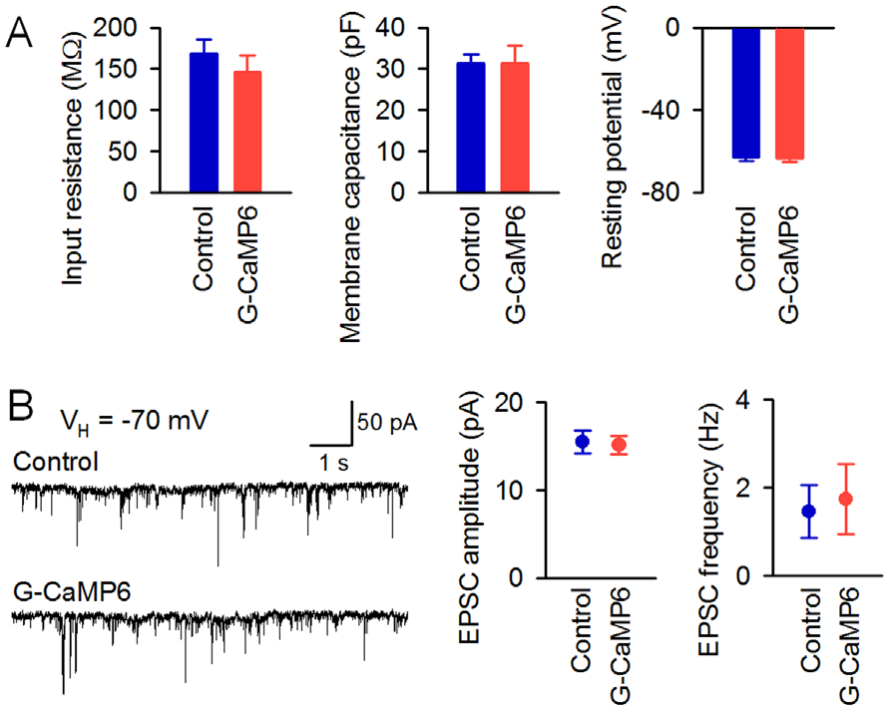
Electrophysiological properties of hippocampal neurons expressing G-CaMP6. **A**, Left, input resistance. Middle, membrane capacitance. Right, resting potential. Error bars, s.e.m. (*n* = 6 each). There were no significant differences between the control and G-CaMP6 groups for any of the parameters (*P*>0.05, Student’s *t*-test). **B**, Left, spontaneous current under the voltage clamp at –70 mV. Middle, amplitude of the excitatory postsynaptic current. Right, frequency of the excitatory postsynaptic current. Error bars, s.e.m. (*n* = 6 each, *P*>0.05, Student’s *t*-test).

By contrast, it was confirmed that the expression of G-CaMP6 does not affect the electrophysiological properties [i.e., input resistance, membrane capacitance, resting potential, excitatory postsynaptic current (EPSC) amplitude, and EPSC frequency] of hippocampal neurons (Fig. 5).

### Imaging of Ca^2+^ Activity in Freely Moving *Caenorhabditis elegans*

**Figure 6:**
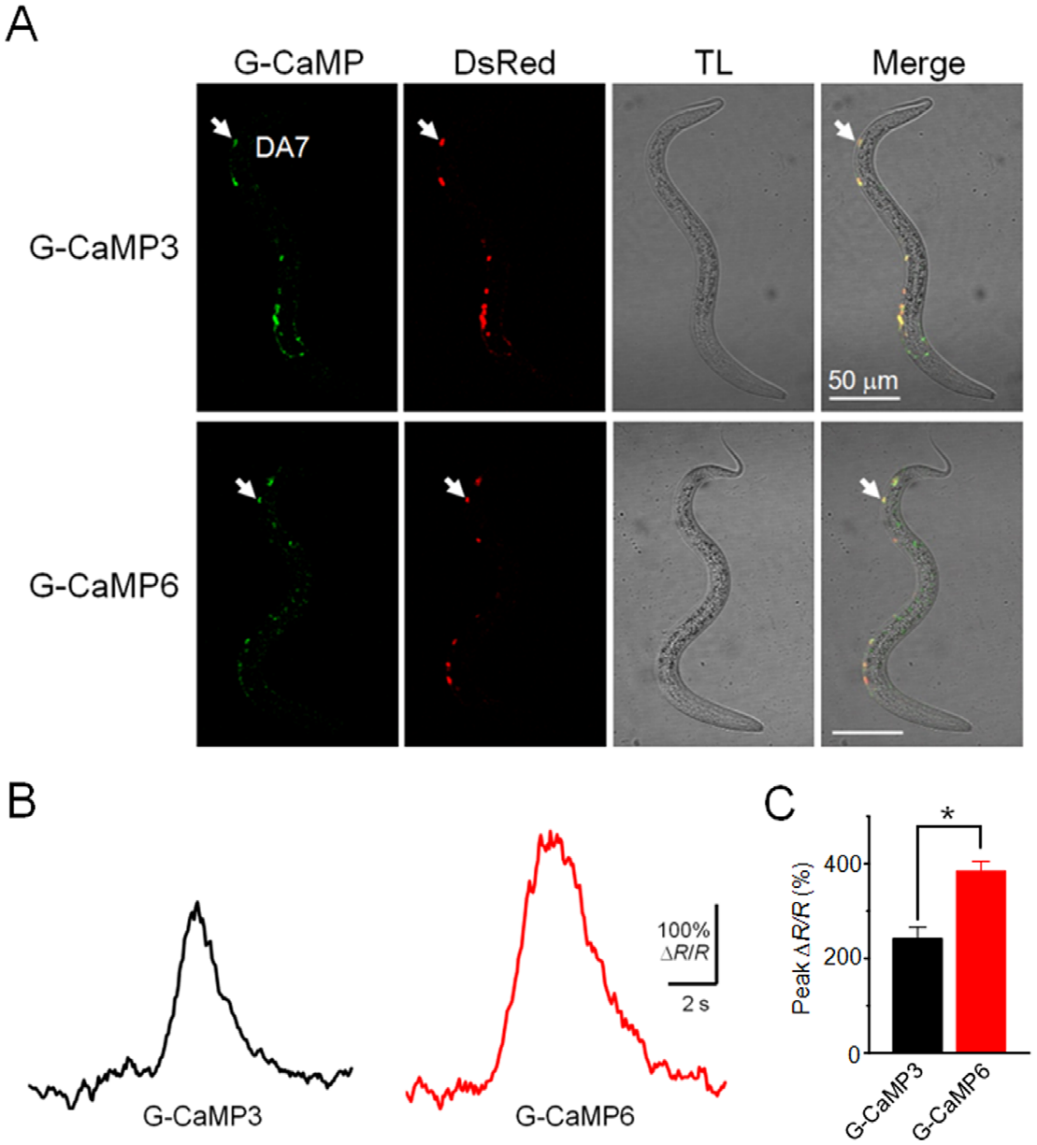
Ca^2+^ imaging of cholinergic DA motoneurons in freely moving *C. elegans*. **A**, Confocal images of L1 larvae expressing G-CaMP6 (*jqEx97*) or G-CaMP3 (*jqEx216*) in the DA motoneurons. In both transgenic strains, DsRed-Express-1 is co-expressed in the DA motoneurons. TL, transmitted-light image. Arrows indicate the DA7 motoneuron analyzed in **B**. **B,** Representative spontaneous fluorescence responses (Δ*R*/*R*) of G-CaMPs from DA7 cholinergic neurons in transgenic worms during locomotion. **C**, Mean peak responses (Δ*R*/*R*). Error bars, s.e.m. (*n* = 10 each from 4 worms, *P = 0.0020, Student’s *t*-test). Movies of the recordings are available as supplementary information (Movies S1 and S2).

We also tested G-CaMP6 in *C. elegans* and successfully recorded spontaneous Ca^2+^ transients in A-type cholinergic motoneurons of freely moving L1 worms. The peak responses (Δ*R*/*R*) of G-CaMP6 during locomotion (Δ*R*/*R* = 3.85±0.20, *n* = 10 from 4 worms) were 1.6-fold greater than those of G-CaMP3 (Δ*R*/*R* = 2.42±0.23, *n* = 10 from 4 worms) (Fig. 6, Movies S1 and S2).

### Imaging of Spine Ca^2+^ Activity with G-CaMP6-actin

**Figure 7:**
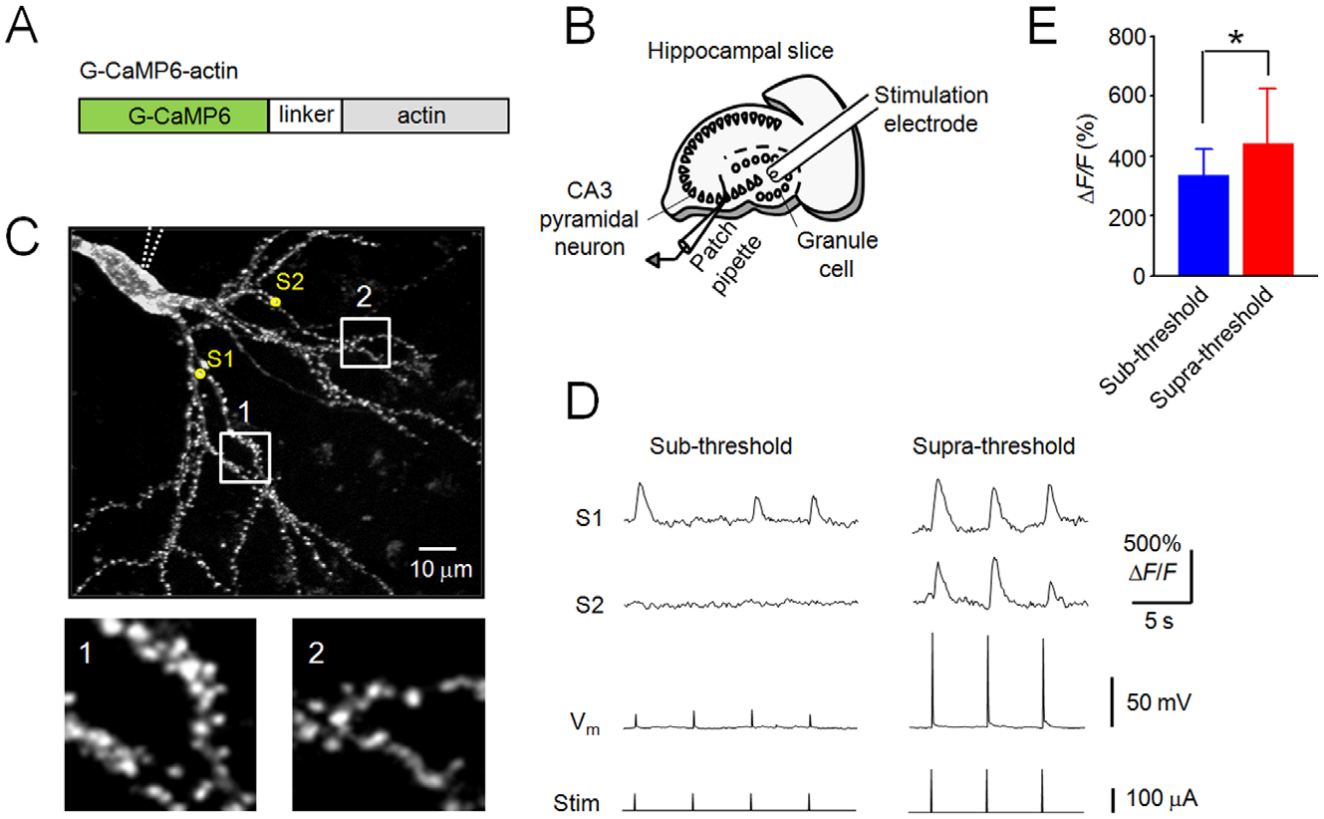
Ca^2+^ Imaging of individual spines in cultured hippocampal slices. **A**, Schematic representation of G-CaMP6-actin. **B**, Schematic drawing of the placement of a stimulation electrode and a patch pipette in a cultured hippocampal slice. **C**, Z-projection of a representative CA3 pyramidal neuron expressing G-CaMP6-actin. The position of the patch pipette is indicated by dotted lines. Two spines of interest (S1 and S2) in the striatum lucidum are indicated by yellow circles. Inset: High-magnification views of rectangular windows. **D**, Changes in fluorescence at S1and S2 and membrane potential (*V*_m_) upon sub- or supra-threshold electrical stimulation (Stim). **E**, Mean responses of active spines. Error bars, s.d. (*n* = 63 and 131 spines for sub- and supra-threshold stimulation, respectively, from 5 slices); *P<0.05, Student’s *t*-test. The average Δ*F*/*F* of the soma in response to supra-threshold stimulation was 15±6.5%.

Next, we targeted G-CaMP6 to dendritic spines, the putative synaptic sites, to reveal the dynamics of individual spine activities. For this purpose, G-CaMP6 was fused with actin, a major cytoskeletal protein within spines, to yield G-CaMP6-actin (Fig. 7A). G-CaMP6-actin was effectively localized to the spines in rat hippocampal CA3 pyramidal neurons (Fig. 7B and C), as has been reported for EGFP-actin and G-CaMP2-actin [8]. We then electrically stimulated the granule cells of the dentate gyrus, which innervate synapses in the striatum lucidum of CA3 region, with signals of two different strengths (Fig. 7B). Intriguingly, the sub-threshold stimulations (Δ*V*_m_ = 18.5±4.8 mV) triggered small fluorescence responses (Δ*F*/*F* = 337±86%, *n* = 256 responses of 63 spines from 5 slices) in a limited number of spines (48.6±6.3%) in the striatum lucidum, with a low response rate in the active spines (57.6±13.8%) (Fig. 7D and E). In contrast, the supra-threshold stimulations triggered large fluorescence responses (Δ*F*/*F* = 443±182%, *n* = 222 responses of 131 spines from 5 slices) in virtually all of the spines in the imaged region including the striatum lucidum and the striatum radiatum, with a 100% activity rate (Fig. 7D and E).

**Figure 8:**
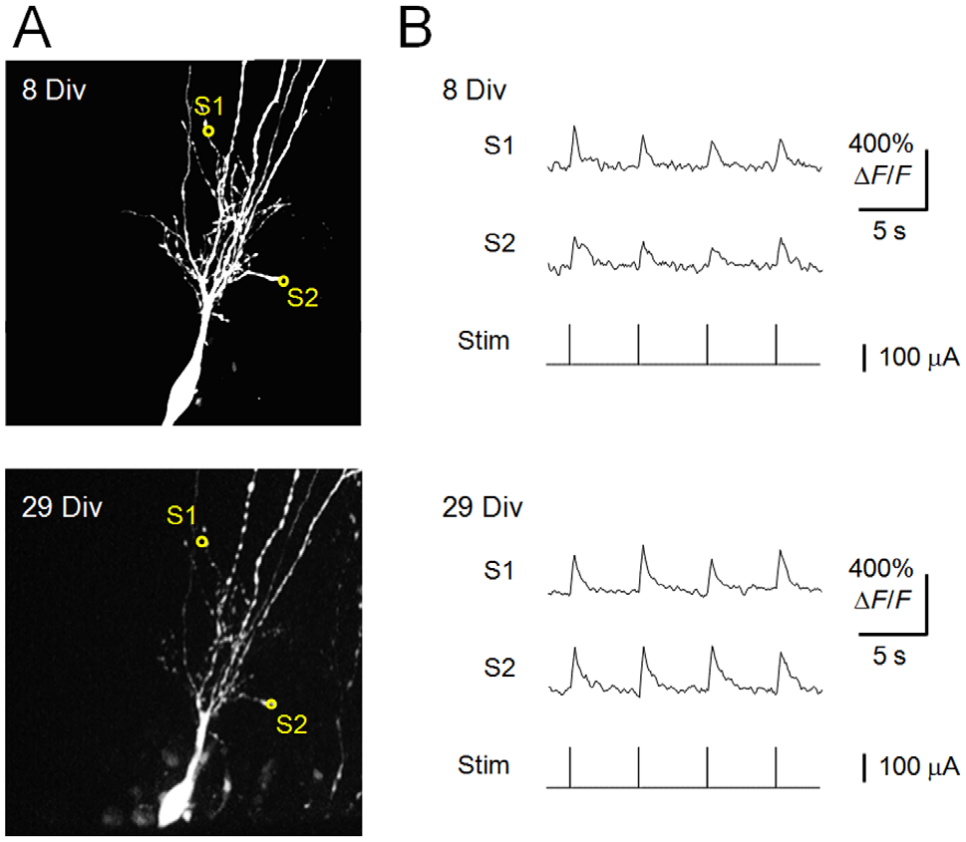
Long-term imaging of Ca^2+^ activity in spines in a cultured hippocampal pyramidal neuron. **A**, Z-projection of a representative CA3 pyramidal neuron expressing G-CaMP6-actin at 8 (upper) and 29 (lower) days *in vitro* (Div). After 7 days *in vitro*, the G-CaMP6-actin plasmid was introduced into the neuron via single-cell electroporation. Two spines of interest (S1, S2) are indicated by yellow circles. **B**, Changes in fluorescence at S1 and S2 upon supra-threshold electrical stimulation (Stim). The average spine Δ*F*/*F* ratios in response to supra-threshold stimulation were 253±30.5% and 201±46.6% at 8 Div and 29 Div, respectively (*n* = 25 spines, *P*>0.05, Student’s *t*-test).

One of the significant advantages of GECIs over chemically synthesized fluorescent indicators is that once indicator genes have been introduced into neurons, the stable expression of the indicator proteins allows long-term recording of the neurons [4], [11]–[13]. To test whether G-CaMP6-actin is applicable to long-term monitoring, Ca^2+^ activity was imaged in spines in slices cultured for 8 and 29 days. After 29 days *in vitro* (Div), the amplitudes of spine Ca^2+^ transients in response to supra-threshold stimulation were not significantly different from those at 8 Div (253±30.5% and 201±46.6% at 8 Div and 29 Div, respectively; *n* = 25 spines, *P*>0.05, Student’s *t*-test). These results confirmed that the expression of G-CaMP6-actin in spines remained stable after at least 4 weeks of culture (Fig. 8).

## Discussion

In this study, we developed high-sensitivity and fast-responsivity GECIs, termed G-CaMP6 and G-CaMP8, by introducing site-directed and random mutations into a prototype GECI, G-CaMP2. Both indicators showed superior performance for reliable detection of neuronal activity with larger fluorescence signals and higher temporal resolution than G-CaMP3. In addition, G-CaMP6-actin captured spine Ca^2+^ dynamics in response to the stimulation of presynaptic afferent fibers.

In the course of developing these superior G-CaMPs, we found three novel mutations for improving the GECI functionality [i.e., ΔH mutation in the RSET domain (in G-CaMP7 and G-CaMP8) and S205N (in G-CaMP7 and G-CaMP8) and I47F (in G-CaMP8) mutations in the circularly permutated EGFP domain]. Based on the G-CaMP2 structure, the residue Ser-205 is located in the β-strand of the circularly permuted EGFP domain (corresponding to the tenth β-strand in EGFP) and facing the inside of the chromophore [18]. This residue has been shown to interact with the chromophore in Ca^2+^-saturated G-CaMP2 [18]. By contrast, the residue Ile-47 is located in the β-strand of the circularly permutated EGFP domain (corresponding to the third β-strand in EGFP) and facing the outside of the chromophore [18]. In addition, this residue is apart from the M13 domain and the CaM domain. Topology of the ΔH position in the RSET domain is unknown, because the available structural analyses of G-CaMPs based on crystallography have been performed using G-CaMP2 without the RSET domain [23] or with another tag [18]. The ΔR2 mutation has been known to enhance the G-CaMP fluorescence in cells by stabilizing the protein [4], but G-CaMP8 bearing this mutation did not show brighter fluorescence than G-CaMP7 in a cellular environment (Fig. 1E and G).

Recently, Akerboom et al. have reported new series of GECIs termed G-CaMP5s [6]. Among these indicators, they have demonstrated that G-CaMP5A, 5G and 5K outperform G-CaMP3 in a wide variety of neuronal preparations. G-CaMP5G, which shows ∼3-fold greater dynamic range (*F*_max_/*F*_min_ = 32.7±1.5) than G-CaMP3 (*F*_max_/*F*_min_ = 12.3±0.4), is the variant which responds with the greatest signals among G-CaMP5s to maximal stimulation when expressed in cultured neurons. Indeed G-CaMP5G is reported to show ∼70% greater signals (Δ*F*/*F*) than G-CaMP3 in response to 1–5 spike trains, but its SNR is not improved with respect to that of G-CaMP3 [6]. Besides, the decay kinetics of G-CaMP5G seems to be almost the same as that of G-CaMP3, judging from the shape of trial-averaged responses of G-CaMP5G and G-CaMP3 (Fig. 2B of [6]). In contrast, G-CaMP8, of which dynamic range (*F*_max_/*F*_min_ = 37.5±3.6) is similar to that of G-CaMP5G, shows ∼100% greater signals than G-CaMP3 in terms of both Δ*F*/*F* and SNR (Fig. 2C and D) and ∼2-fold more rapid decay kinetics than G-CaMP3 (Fig. 2E). On the other hand, a drawback of G-CaMP8 is its dim baseline fluorescence in neurons, which needs to be improved in the future. G-CaMP5K is the most sensitive G-CaMP5 variant (*K*_d_ = 189±5.0 nM) [6] and is likely to be useful for detecting small neuronal Ca^2+^ signals, similar to G-CaMP6 (*K*_d_ = 158±4.0 nM)(Fig. 1B and C). G-CaMP5K is reported to show ∼2-fold greater signals (Δ*F*/*F* and SNR) than G-CaMP3 in response to 1–5 spike trains [6]. G-CaMP5A is the variant with intermediate sensitivity (*K*_d_ = 307±12 nM) and signal amplitudes (*F*_max_/*F*_min_ = 17.4±1.2) among G-CaMP5s, but is reported as the preferred variant over G-CaMP5G and G-CaMP5K for use in worm and zebrafish [6]. It is good for researchers to have the option to select the ideal GECI depending on their own applications. Because new G-CaMPs (G-CaMP6 and G-CaMP8) and G-CaMP5s have been optimized by different strategies, it may be possible to combine the mutations in the different sets of G-CaMPs to further improve them.

The detection of neuronal activity patterns with single-spike resolution is required to elucidate neural network dynamics. We demonstrated that G-CaMP6 and G-CaMP8 faithfully detected Ca^2+^ transients in response to single spikes in pyramidal neurons in hippocampal slices at 25–28°C. However, it is still unknown whether these G-CaMPs exhibit similar performance *in vivo*. As shown in Fig. 4, both the dynamics of intracellular Ca^2+^ and the sensitivity of Ca^2+^ indicators are temperature dependent. Indeed, it has been reported that GECI fluorescence is less intense *in vivo* compared to *in vitro* [4], [24]. Another point to note is that the detectability of indicators might be affected by the expression levels of indicator proteins. Therefore, further studies are needed to determine whether similar results can be obtained in the other gene expression systems, such as transgenic mouse lines or viruses. The decay time constant of spike-induced Ca^2+^ transients of the newly-developed G-CaMPs ranged between 400 and 450 ms, which is shorter than that of G-CaMP3 [4]. We demonstrated that the rapid kinetics of Ca^2+^ indicators contribute to discrete fast individual spikes in burst-spike trains with a temporal resolution of up to 15 Hz. To our knowledge, G-CaMP6 is the most suitable GECI currently available for detecting and isolating fast individual spikes in spike trains.

Excitatory synaptic activity induces a transient Ca^2+^ increase in individual spines through the activation of voltage-sensitive Ca^2+^ channels and/or NMDA receptors. In previous studies, spine Ca^2+^ activity was imaged with synthetic indicators, such as Oregon Green BAPTA-1 [25], [26]. In fly neuromuscular junctions, postsynaptically targeted G-CaMP2 (SynapG-CaMP2) has been reported to respond to excitatory postsynaptic currents [9]. In mammalian cells, Mao et al. [8] developed G-CaMP2-actin to record Ca^2+^ signals within spines but failed to detect synaptically evoked Ca^2+^ activity, presumably because of the low Ca^2+^sensitivity of G-CaMP2. In this study, we demonstrated that G-CaMP6-actin is the first GECI that allows the visualization of Ca^2+^ signals in response to synaptic stimulation at the single-spine level. Although the exact mechanisms of spine Ca^2+^ signals remain unknown, it seems likely that sub-threshold stimulation triggers Ca^2+^ transients through postsynaptic NMDA receptors [27], while supra-threshold stimulation triggers Ca^2+^ transients at 100% of the spines by opening of voltage-gated Ca^2+^ channels through backpropagation of action potentials. In principle, we should be able to visualize spine responses to evaluate long-term plasticity, which is thought to be an elementary component of learning and memory. We expect that these novel G-CaMP technologies, together with advanced imaging systems [26], [28], will facilitate our understanding of neuronal network dynamics in the brain at the single-synapse level.

## Materials and Methods

### Plasmid Construction

Complementary DNAs (cDNAs) encoding sfG-CaMP2, sfG-CaMP2.02, G-CaMP5.09 and G-CaMP6 were synthesized by mutagenizing the cDNA encoding the prototype GECI, G-CaMP2 [15], using the QuikChange Lightning Multi Site-Directed Mutagenesis Kit (Agilent). cDNAs encoding G-CaMP7 and G-CaMP8 were synthesized by randomly mutagenizing the cDNAs encoding G-CaMP6 and G-CaMP7, respectively, as previously described [15]. The cDNA encoding G-CaMP3 was constructed by introducing mutations [4] into the G-CaMP2 cDNA. These cDNAs were subcloned into a pRSET_B_ vector (Invitrogen) containing a T7 promoter, as described [15] for bacterial expression, or into a pEGFP-N1 vector (Clontech) with a CMV promoter, as described [3] for expression in HeLa cells and cultured rat hippocampal neurons. For *in utero* electroporation, cDNAs encoding G-CaMPs and mCherry (Clontech) were subcloned into a pCAGGS vector containing a CAG promoter (CMV enhancer, β-actin promoter and woodchuck hepatitis virus regulatory element [WPRE]) [4]. To target G-CaMP6 to dendritic spines in neurons, a G-CaMP6-actin indicator was generated by fusing a cDNA encoding human β-actin (derived from pAcGFP1-acin, Clontech) to the 3′ end of a cDNA encoding G-CaMP6 via a linker encoding the amino-acid sequence GGGTGGSRSRARGTVDCRIRSLSSRSRA (in one-letter code). To generate plasmids to express G-CaMPs in the DA motoneurons in *C. elegans*, cDNAs encoding G-CaMPs were subcloned into a pFX_EGFPT vector containing the *unc-4* promoter [29]. All of the constructs were verified by sequencing.

### Bacterial Protein Expression and *in vitro* Characterization

*E. coli* KRX (Promega) transformed with pRSET_B_-G-CaMP was grown at 37°C, and protein expression was induced by adding 0.1% rhamnose and incubating for an additional 5 h at 20°C. The indicator proteins with N-terminal histidine tags were purified, dialyzed against KM buffer containing (in mM) 100 KCl and 20 MOPS (pH 7.5) and used for *in vitro* characterization [15]. Spectral analyses were performed as previously described [16], [17]. The term “dynamic range” was defined as *F*_max_/*F*_min_, where *F*_max_ is the fluorescence intensity at saturating [Ca^2+^], and *F*_min_ is the fluorescence intensity at nominally zero [Ca^2+^] with 1 mM EGTA. The Ca^2+^ titration experiments were performed at pH 7.2 with 10 mM solutions of K_2_H_2_EGTA and Ca_2_EGTA from the Ca^2+^ Calibration Kit #1 (Invitrogen), as previously reported [30].

### Ca^2+^ Imaging in HeLa Cells

HeLa cells were cultured in Dulbecco’s modified Eagle’s medium containing 10% fetal bovine serum and transfected with plasmids using Lipofectamine 2000 (Invitrogen) according to the manufacturer’s manual. Fluorescence images of cells expressing G-CaMPs were acquired with a fluorescence microscope (IX71, Olympus) equipped with a CCD camera (ORCA-ER, Hamamatsu), as previously described [16], [17]. The cells were perfused with HEPES-buffered saline (HBS) containing (in mM) 135 NaCl, 5.4 KCl, 2 CaCl_2_, 1 MgCl_2_, 10 glucose and 5 HEPES (pH 7.4), and after reading the baseline fluorescence, 100 µM ATP was bath-applied for 1 min. The images were analyzed using AquaCosmos version 2.0 software (Hamamatsu). The transient increase in fluorescence (Δ*F*/*F*) was calculated after subtracting the background fluorescence.

### Ca^2+^ Imaging in *C. elegans*

The expression plasmid carrying G-CaMP6 (*Punc-4::G-CaMP6*) or G-CaMP3 (*Punc-4::G-CaMP3*) was co-injected with the plasmid carrying DsRed-Express-1 (*Punc-4::DsRed-Express-1*) [29] into wild-type N2 worms using a standard protocol [31]. The *jqEx97* (G-CaMP6) strain and the *jqEx216* (G-CaMP3) strain were used in this study. Ca^2+^ imaging was performed in worms on a 1.5% agar pad placed on a glass slide (76×26 mm, 1.0- to 1.2-mm thickness, Matsunami). L1 animals were placed in M9 buffer [32] and dropped onto the agar pad, and the glass slide was covered by a cover glass (24×24 mm, 0.12- to 0.17-mm thickness, Matsunami). The worms were then subjected to imaging analyses using an A1R laser confocal microscope (Nikon) and NIS-Elements AR 3.2 image acquisition software (Nikon). The images were captured with manual movement of the X and Y positions of the stage to track the worms. Confocal images (512×512 pixels) of cholinergic DA motoneurons were captured at 15 frames per second (fps) with a water immersion objective (40×, 1.15 NA, Nikon). After the subtraction of background noise, the fluorescence ratio changes (Δ*R*/*R*) of G-CaMP6 or G-CaMP3 against DsRed-Express-1 were calculated as (*R*_1_–*R*_0_)/*R*_0_, where *R*_1_ is the fluorescence ratio at any time point and *R*_0_ is the baseline fluorescence ratio.

### Cultured Slice Preparation and Single-cell Electroporation

All experiments were performed with the approval of the animal experiment ethics committee at the University of Tokyo (approval number: 19–43) and according to the University of Tokyo guidelines for the care and use of laboratory animals. Hippocampal slices from postnatal day 7 Wistar/ST rats (SLC) were prepared, as previously described [33], according to the guidelines for laboratory animal care and safety of the University of Tokyo. Briefly, rat pups were chilled with ice and decapitated. The brains were removed and cut horizontally into 300-µm slices using a DTK-1500 vibratome (Dosaka) in aerated, ice-cold Gey’s balanced salt solution supplemented with 25 mM glucose. The entorhino-hippocampal stumps were excised and cultivated on Omnipore membrane filters (JHWP02500, Millipore) that were laid on plastic O-ring disks. The cultures were incubated in a humidified incubator at 37°C in 5% CO_2_ with 1 ml of 50% minimal essential medium, 25% Hanks’ balanced salt solution (HBSS), 25% horse serum (Cell Culture Laboratory) and antibiotics. The medium was changed every 3.5 days. On days 3–5 *in vitro*, G-CaMPs and mCherry under the control of the CMV promoter were introduced into the neurons via targeted single-cell electroporation [20]. Briefly, borosilicate glass pipettes (tip resistance, 5–7 MΩ) were filled with HBSS containing 1–2 µg/µl of plasmid DNA. After the tip of the pipette was placed in close proximity to the soma, electroporation was performed with 50 rectangular pulses (–5 V, 0.5-ms duration) at a frequency of 50 Hz. Single-cell electroporation was applied sequentially to up to 10 cells using the same pipette within 5 min. Imaging was performed 24–48 h after electroporation.

### *In utero* Electroporation and Acute Slice Preparations

Day-14-pregnant ICR mice (CLEA Japan) were deeply anesthetized, and their intrauterine embryos were removed surgically, as previously described [21]. To express G-CaMPs and mCherry, expression plasmids under the control of the CAG promoter (2 µg/µl) were injected into the lateral ventricle of the intrauterine embryos, and electric pulses (33 V, 50 ms, 4 times) were applied through a CUY650P5 forceps-type electrode using a CUY-21 electroporator (Nepa gene). At postnatal days 10–16, the transformed mice pups were anesthetized with ether and decapitated, and their brains were immersed in ice-cold artificial cerebrospinal fluid (aCSF) consisting of (in mM) 27 NaHCO_3_, 1.4 NaH_2_PO_4,_ 2.5 KCl, 7.0 MgSO_4_, 1.0 CaCl_2_, 0.5 ascorbic acid and 222 sucrose, bubbled with 95% O_2_ and 5% CO_2_. Coronal cerebral hemispheric slices (400-µm thick) were cut using a Vibratome 3000 (Vibratome).

### Electrophysiology and Ca^2+^ Imaging in Cultured Hippocampal Slices

Hippocampal slices were mounted in a recording chamber and perfused at a rate of 1.5–3 ml/min with aCSF containing (in mM) 127 NaCl, 26 NaHCO_3_, 3.3 KCl, 1.24 KH_2_PO_4_, 1.0 MgSO_4_, 1.0 CaCl_2_ and 10 glucose, bubbled with 95% O_2_ and 5% CO_2_. All recordings were performed at room temperature (24–28°C), unless otherwise specified. Patch-clamp recordings were collected from hippocampal CA3 pyramidal neurons using a MultiClamp 700B amplifier and a Digidata 1440A digitizer controlled by pCLAMP10 software (Molecular Devices). Epifluorescence microscopy was used to select cells showing stable mCherry expression with a fluorescence intensity ranging from 103 to 125 (arbitrary units). Borosilicate glass pipettes (5–7 MΩ) were filled with a solution containing (in mM) 135 K-gluconate, 4 KCl, 10 HEPES, 10 phosphocreatine-Na_2_, 0.3 Na_2_-GTP and 4 Mg-ATP (pH 7.2). The signals were low-pass filtered at 1–2 kHz and digitized at 20–100 kHz. Data were discarded if the access resistance changed by more than 20% during the experiment. Spikes were evoked by current injections (2–3 ms, 1–2 nA), and electrical stimulation was applied using a constant voltage-isolated stimulator and a glass pipette filled with aCSF (Nihon Kohden). The electrodes were placed in the dentate hilus to stimulate the mossy fiber pathways. For sub-threshold stimulation, the stimulation intensity was adjusted so that <50% of the spines exhibited Ca^2+^ transients (40–120 µA, 50 µs). For supra-threshold stimulations, the intensity was raised to more than 200 µA so that almost all neurons generated action potentials. For the Ca^2+^ imaging, the G-CaMPs were excited at 488 nm with a laser diode (641-YB-A01, Melles Griot) and visualized using a 507-nm long-pass emission filter. Images were captured at 50–500 fps using a Nipkow-disk confocal scanner unit (CSU-X1, Yokogawa Electric), a cooled CCD camera (iXON DV897, Andor), an upright microscope (Eclipse FN1, Nikon) and a water-immersion objective (40×, 0.9 NA, Nikon). The cell bodies of the neurons were carefully identified by eye to locate regions of interest (ROIs). Cells with labeled nuclei were excluded from further analysis because both Ca^2+^ homeostasis and G-CaMP function are impaired in these cells [4]. In each ROI, the fluorescence intensity was spatially averaged. The fluorescence change was defined as Δ*F*/*F* = (*F_t_* – *F*_0_)/*F*_0_, where *F_t_* is the fluorescence intensity at time *t*, and *F*_0_ is the baseline averaged for 2 s before time *t*. The maximum Δ*F*/*F* within 1 s after action potential initiation was used a measure of the peak amplitude of the Ca^2+^ transient. The SNR was defined as the average spike signal amplitude divided by the standard deviation of the fluorescence intensity in the trace. Data were collected from >3 consecutive trials. The rise time τ_1/2_ was measured as the time between the onset of the spike initiation and the half-peak response. The decay time τ_1/2_ was measured as the time of half decay of a single exponential fit of the recovery from the peak response to the baseline. For spine Ca^2+^ imaging, response rates were measured in the spines that exhibited at least one Ca^2+^ transient during imaging period (i.e. active spines). The rates were defined as the ratio of the number of Ca^2+^ transients to the total trial of electrical stimulation.

## Supporting Information

Movie S1 **L1 larvae (** ***jqEx97*** **) co-expressing G-CaMP6 and DsRed-Express-1 in the DA neurons during locomotion (corresponds to** **Fig. 6** **).** Green, red and transmitted-light images were overlaid. Images were taken at 15 fps.
(AVI)

Movie S2 **L1 larvae (** ***jqEx216*** **) co-expressing G-CaMP3 and DsRed-Express-1 in the DA neurons during locomotion (corresponds to** **Fig. 6** **).** Green, red and transmitted-light images were overlaid. Images were taken at 15 fps. A worm was kept in the imaging field by manual adjustment of the x-y stage.
(AVI)
